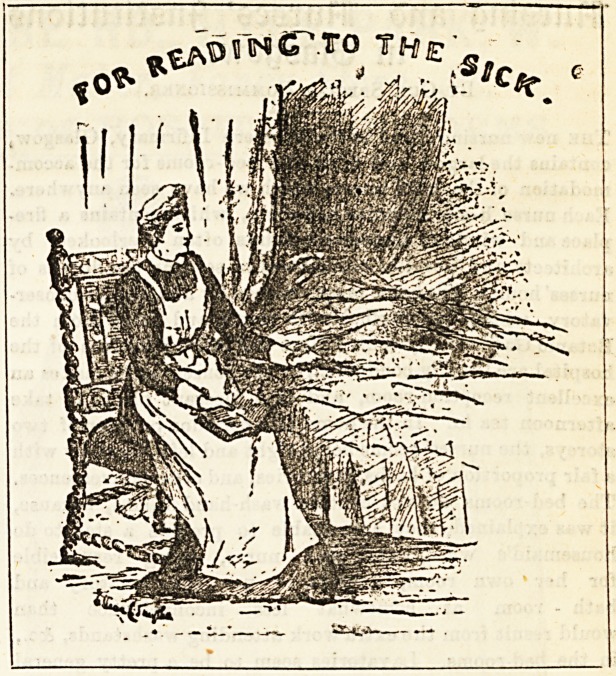# The Hospital Nursing Supplement

**Published:** 1893-01-21

**Authors:** 


### "The Hospital" Nursing Mirror.

Being the Extra Nursing Supplement of "The Hospital" Newspaper.

[Contributions for this Supplement should be addressed to the Editor, The Hospital, 140, Strand, London, W.C., and should have the word "Nursing" plainly written in left-hand top corner of the envelope.]

### News from the Nursing World.

### NURSES' CO-OPERATION.

Her Royal Highness Princess Mary, Duchess of Teck, has graciously consented to preside at the second annual meeting of the Nurses' Co-operation, at four p.m., on Thursday, February 2nd, at 20, Hanover Square. Tickets can be obtained on application to the Lady Superintendent, 8, New Cavendish Street, W.

### GOOD-BYE, CHRISTMAS!

Whatever Boards of Management may think of the financial position of their hospitals, the general public is unanimously agreed that the last Christmas is one of the pleasantest on record, alike for patients and nurses. Much space has been given in The Hospital to seasonable subjects, and now other matters claim attention. In bidding Christmas a friendly farewell, readers are counselled at the next anniversary to send in their reports of their festivals as early as possible.

### LONDON'S END.

Under this heading an article appeared in The Hospital last September, describing that outlying district known as Plaistow, in Essex. One hundred thousand poor inhabitants necessarily include many sick persons, needing not only nursing but food. St. Mary's Home supplies between thirty and forty nurses at present; three years ago there were only two. Miss Katherine Twining has recently established a kitchen, where diets suitable for sick people are prepared and distributed. A greater boon to deplorably destitute invalids cannot well be imagined, and gifts in kind as well as in money are asked for immediately. To persons in a position to give, a more practical outlet for their charity cannot easily be conceived.

### A LASTING MEMORIAL.

When the late Mrs. Wardroper retired from St. Thomas's Hospital, the probationers then in training joined with a number of the former "Nightingales" in giving her a testimonial. About one hundred and fifty nurses, valuing the advantages secured to them by her efforts, showed their gratitude in an address, which was accompanied by a handsome tea and coffee service. Doubtless so unanimous a tribute was deeply appreciated by Mrs. Wardroper; and now it seems fitting that a lasting memorial should be raised in her name. The form most suitable for this, perhaps, needs discussion, but it will be a proof that thousands of trained nurses owe directly or indirectly their knowledge and their honourable independence to one matron's noble life-work. If Miss Nightingale's name stands first in the hearts and the minds of qualified nurses then Mrs. Wardroper's should certainly rank second.

### PRESENTS AND PRESENTATIONS.

So-called Presentations were formerly made exclusively to such nurses as distinguished themselves, either by special acts of heroism or by long and faithful service. In the first case, no doubt existed as to the justice of the offering, nor could there be aught save satisfaction at a complimentary gift made to a matron or nurse leaving her hospital with a record of good work therein accomplished. But what value can be attached to the modern unmeaning and unending presentations which have become fashionable, and even, in a certain way, competitive? They are, in fact, in numerous instances, a heavy annual tax, which women workers can ill afford to pay. A small present to a superintendent from a nurse who has ceased to work for her is a valued proof of affection; but gifts made to present employers are capable of another and baser interpretation. The motive of a presentation to a person on the eve of departure admits of no criticism, but this cannot invariably be said of other varieties of giving. The modern subscription list in the nurses or servants' refectory is an unwelcome bugbear to many a liberal-minded nurse and maid-servant of a limited income with unlimited calls made upon it.

### REST AND FRESH AIR.

The subject of suitable holiday resorts seems to pass out of sight in the winter months, but it ought not to do so. Nurses and other busy people require occasionally "a few days off duty" at all seasons of the year. The long strain of a heavy case makes a week of quiet rest essential for the wearied attendant, and the question of where to go has to be faced without regard to the calendar. If a nurse wants plenty of fresh air, unlimited quiet, clean, moderate lodgings, and any number of pretty walks, she cannot do better than take a train to Seaford, in Sussex. After two or three strolls, long or short ones, according to taste, over the beautiful downs, which are practically a part of the quiet little town, a visitor begins to think that life is not only worth living, but means also enjoyment. People forget to be tired in the clear, keen air, and there is always sunshine and blue sky to be seen during part of the day. If anyone wishes to spend money and to live in a crowd this end can be achieved by visiting Seaford in August, but should a tired nurse want to regain health and vigour at an economical rate of expenditure she can realize her wishes by going there during the other months of the year.

### PLEADING FOR TRAINED NURSING.

In the *Times of India* our friend "Thomas Atkins" has been pleading earnestly for trained nurses to be supplied to outlying districts. He thinks, fairly enough, that it is hard upon serious cases to be entirely in the hands of orderlies, simply because the sufferers are at uninteresting stations. He speaks, somewhat bitterly, of Poona and Mhow absorbing all the nurses sent to the Presidency; and he seems to fear that the promised increase in the numbers supplied, will benefit those stations only. We hope his forebodings are without foundation, for, as he says, such a step would be "a manifest injustice to outlying stations," and certainly the fact of places being "very pleasant to be quartered in, and very desirable socially," gives them no claim to an undue proportion of those ladies whose services are demanded and appreciated by the British soldier in India.

### MEDICINE WITH OR WITHOUT NURSING?

THE number of hospitals in India is steadily increasing, and a new one, which was named "Rani Raghuraj Koer Lady Dufferin," was lately opened at Partabgarh by Sir Auckland Colvin. But more hospitals must not be taken to mean a greater demand for trained English nurses, as we are told that a lady doctor with Eurasian and native nurses constitute the usual staff. It would be interesting to know something of the qualifications of these so-called nurses, particularly as to the extent of their experience and knowledge. Lady doctors are rarely themselves trained nurses, and, moreover, many of them have entered on their medical studies at too early an age to secure previous acquaintance with general management or organisation. Those conversant with the absolute necessity for trained superintendents in other countries, will endorse our opinion that the Indian Empire will have no assured hospital reputation unless the labours of women doctors are assisted by adequately-trained matrons. The hospital appointments of lady doctors are not well paid in India; women make better incomes in private practice. On completion of their five years of service, Acting Superintendents E. M. Lickfold and M. E. M. Latch, with Nursing Sister A. Watkins, will be permitted to retire from the I.N.S., and Nursing Sisters E. C. Kayes and M. Carlisle have also received official consent to their retirement. Nursing Sister Hammans, who has been stationed at Quetta since she joined the I.N.S. at the end of 1891, has recently obtained the post of Superintendent-Matron at Madras. This appointment is a civil one, and the hospital is one of the finest in India, containing many modern improvements, and the matron's salary is a very good one, but of course the climate of Madras is somewhat trying to Europeans.

### UNTRAINED, THOUGH KINDLY.

THE nursing at the fine General Civil Hospital at Colombo is conspicuous by its absence. The patients are attended to kindly by a Roman Catholic Sisterhood, whose members are absolutely untrained, native men and women doing the rougher parts of the service. An Anglican community offered to send out nurses for this hospital, but unluckily the previous offer of the other Sisterhood had already been accepted. Even the present kindly, untrained supervision is only partial in character, for night watching is trusted entirely to certain natives, who are known as night orderlies. Irish nuns are, therefore, not the only ladies who consider the sick can dispense with their services during the weary hours of the night. There are excellent medical men in Ceylon, the comprehensive title of "European Doctor" being applied to men of all nationalities and colours who have obtained their "qualifications to practice" in Europe. The "native doctor" is an "unqualified practitioner" in the completest significance of the term. But the men possessed of European knowledge and experience must feel frightfully handicapped when their own abilities are unsupported by the assistance of the trained nurses, whose aid is considered absolutely indispensable by English medical men.

### NURSING BY DEACONESSES.

THE nursing at the "Charité" Hospital, in Berlin, where there are 2,000 patients, is undertaken by various orders of deaconesses. Each class of cases, such as medical surgical, male, female, or children, being under separate supervision. Every order has an "Oberin" (sister-in-charge), and she is directly responsible to the Medical Superintendent. There are also a few ordinary nurses, unattached to any Sisterhood, who, having been for some years at the hospital, have got into the way of doing certain parts of the work. The whole system is said to work smoothly and well. The wards are clean-looking but very bare, as there is no attempt at adornment.

### PARISIAN PETS.

MANY graceful novelties are transferred annually from Paris to London, but the last new nursing sensation of that gay city will hardly find universal imitation. The Town Council of Paris has decided to give a grand reception to matrons and nurses who "were on duty during the last cholera epidemic." Truly "duty" is a word which has changed its significance if such entertainments be required. Doubtless people have a dread of cholera, and in the East it is the sole disease feared by the natives, its victims not even receiving the usual heathen obsequies. But intelligent European women run no greater personal risk in nursing cholera than in attending on fevers, including small-pox. Neither are the incessant labours of district nurses undertaken without daily danger, whilst the attendants in casualty and out-patient departments of general hospitals are liable to hourly contact with all varieties of infectious diseases. If these and many other brave and unselfish women neither desire nor obtain fetes in their honour, why should such be accorded to nurses who did simply their duty during one short-lived epidemic?

### A DISABLED NURSE.

ALL subscriptions received from friends for the nurse who injured her back in the discharge of her duty will be acknowledged each week in THE HOSPITAL. We have received the following this week: Nurse Louie, H. F. G., and E. F. L., each send a shilling; M. J. Harris, 2s.; Nurse Meyer, 3s. 6d.; No. 1,281, 2s.; Mrs. M. Minet, £2; C. H. M., 5s.; and H. L. W., 5s. Each offering is accompanied by a letter expressing warm sympathy and kindly interest in our invalid sister.

### SHORT ITEMS.

MISS HONNOR MORTON has kindly consented to resume the duties of Hon. Secretary to the Nurses' Co-operation in New Cavendish Street.—Nurse Jefferd has received Hale's "Art of Massage" for her prize, and Miss Anderson has been awarded "The Art of Feeding the Invalid" for best answers to Examination Questions for November and December respectively.

### The Development of Children by Gymnastics.

V.

HAVING touched on some of the exercises with the chief moveable apparatus, a series which is embraced generally under the name of "calisthenics," I should like to say a few words about the more advanced course practised in the gymnasium proper. For these larger, and in many cases fixed, machines are necessary, and the movements executed on or with them require a certain former training, supplied either by calisthenics or at least by some natural course, as vigorous daily exercise in the form of walking, running, jumping or such-like brisk habits. Of course the number of children who are enabled to benefit by regular attendance at a gymnasium is proportionately small to those who may use poles, dumb-bells, or even Indian clubs, but we hope their ranks may rapidly swell with realisation that a vigorous, healthy training turns our boys into men and our girls into worthy mothers of men; while neglect of such training is likely to produce cowardly sneaks and specimens of a contemptible wavering character, who, if they fall into unprincipled hands, are apt to commit blunders, if not crimes, greater even than those of the resolute villain.

The room set apart for the gymnasium should be large, and above all things well ventilated. This is essential, as one of the most important objects of vigorous exercise is to quicken the organs of respiration, and, this gained, the full benefit can only be felt when the air inspired is pure, a result only obtainable in rooms, where any number of people are collected, by a free, rapid, and constant change of atmosphere.

When funds and space are available a perfect combination is effected by the addition of a swimming bath; for some of the healthiest essentials of the body are supplied by the daily "header," and the muscles of the arms and legs gain tone and density from the strong stroke of a good swimmer, while the chest is expanded, thus stamping out one of the primary tendencies to consumption in delicate children.

Much of the gymnasium apparatus is merely used for the sake of varying exercises of the same character, making modifications as to difficulty; for instance, the inclined plank, ladder, and pole are only variations in the same machine, and the same movement made on each in succession increases in difficulty according to the apparatus. The horizontal beam is one of the first used. It is a stout wooden pole some ten or twelve inches in diameter, and supported firmly at either end, and raised at first a short distance from the ground, the height being greater as the pupil gains confidence and skill. The chief object of the exercises on this is to promote easy balance and secure equilibrium of the body under circumstances which might chance to present themselves through life. It also gives a firmness to the step, and erectness of head and body, which ought to mark a straightforward, noble character. A somewhat similar apparatus is the horizontal bar; but this is used for a series of vaulting, leaping, and arm-balancing exercises, and these are, in their turn, extended by others on another machine known as the "vaulting horse." This last is a rounded wooden "body," representing in form the "trunk" of a horse's body, and is well padded in the centre, and the whole supported on firm legs. The almost numberless forms of exercises connected with "the horse" are chiefly calculated to develop the muscles of the arms and strengthen the trunk, thus being most valuable in invigorating the digestive organs. It is interesting to observe the great disparity of muscular development in leg and arm organism. In infancy a child will clutch the nearest object, and often raise itself by its arms; but as it acquires the art of walking, the proportion of labour assigned to the upper limbs becomes almost infinitesimal in comparison done by the lower members; but the material for development is not wanting, and this surely points to the fact that we are intended to make more use of them than we do at present.

ADJUSTABLE APPARATUS.

Felix Oswald, in his excellent work on physical education, refers to the subject: "Taking a step means to support and propel, or even lift, the whole body by means of the foot remaining on the ground. In running up and down stairs, to school and back, and here and there about the house, the legs of the laziest schoolboy performs that feat about eight thousand times a day. What have his arms done in the meanwhile? Carried a chair across the room, perhaps, or elevated so and so many spoonfuls of hash from a plate to a place six inches further up. . . . To equalise this difference should therefore be the primary object of physical culture, for the harmonious structure of all its parts is an essential condition of a perfectly developed body." It is then mainly with this object in view that we advocate the use of the vaulting horse and other arm-developing exercises, such as the trapezium and parallel bars, and we give an illustration of a simple apparatus which may be adjusted to various heights suitable for children, and used for some of the easier exercises of this class.

Now that Alpine climbing has become all the rage, it is hardly necessary to advocate, for boys at least, a regular training in what is called "wall" climbing, a practice of ascending and descending a perpendicular plane by means of small grooves cut, at intervals, in it. This and the more ordinary "ladder" and "rope" climbing will, if possible, raise the feats of the future Alpine Club members to a level exceeding that of their fathers.

In alluding to the above exercises we have not followed the progressive course taken in the gymnasium, but we hope that it will not therefore be supposed that such an one is wanting. A good gymnastic instructor will graduate his exercises to the increasing strength and efficiency of his class, and, though the Lyng system is perhaps more avowedly drawn up on strict lines of progression, yet there is a gradually advancing course which is followed in every gymnasium under the direction of a really capable manager.

### Examination Question for December.

You are nursing a patient with rheumatic fever. The doctor has paid his daily visit, and lives miles away. In the afternoon you find the sweating has ceased, there is slight delirium, and the temperature is 106 deg. What would you do?

In the majority of answers received to this question we are sorry to find all reference to the doctor is omitted. Three nurses boldly prescribe drugs, and we shudder to think of the probable results of such doses as antimony, diaphoretic powder, antipyrin, aconite, and paragoric in the hands of unqualified persons. One nurse carefully describes sponging and the cold pack, but we pity the poor patient whose sheet is changed "every three minutes, or oftener!" Another nurse dictates an ice bath, which is certainly heroic treatment in so critical an illness. One competitor fully explains that it is a nurse's duty to obtain previous orders from the doctor in attendance, but she leaves us in ignorance of her own proposed line of action in an emergency. Three answers are good, and we give the one sent by E. A. Anderson, to whom the prize is awarded :—

I should at once proceed to sponge the patient with tepid water, but if this did not reduce the temperature or stop the delirium, I should use the "wet pack." At the same time, feeling that the sudden rise of temperature and the other symptoms are likely to be the onset of one of the complications of rheumatic fever, pericarditis, pneumonia, &c., I should send for the doctor, acquainting him with the measures I had taken.

EXAMINATION QUESTION FOR FEBRUARY.

What precautions against infection should be taken during and after the nursing of a case of scarlet fever in a private house?

Answers must be sent in by February 3rd. They must be concise, but thorough, written on one side of the paper only, accompanied by writer's name and address, and directed to "Nursing," Editor of THE HOSPITAL.

### Grange Convalescent Hospital.

THIS is a small Convalescent Home at Torquay for the use of fourteen patients, and its charges for the admission of men and of boys over fifteen years of age are most moderate, namely, eight shillings a week. There are few rules, and those there are, are necessary ones. Any person in London desiring admission must have a certificate from his doctor stating the nature of his complaint, and this certificate must be examined and countersigned by Dr. L. W. Sedgwick. Chronic and incurable cases are not admitted. The Matron is a trained nurse, and the patients, we are glad to say, get a real rest, and do not help in any way in the work of the home, and are exempt even from cleaning their own boots! It is always a source of great satisfaction to us to hear of a home of this sort, not hemmed in by unnecessary rules and regulations and subscribers' letters, and we feel that many of our readers will share this feeling. Applications must be made to the Hon. Helen Cubitt, Lyncourt, Torquay.

### Everybody's Opinion.

### GIRL NURSES.

"A THOUGHTFUL WOMAN" writes: I saw a very good article on "Girl Nurses" in THE HOSPITAL some weeks ago, and I hoped that it would have been followed up by others. It is a most serious subject, and one that comes home to all experienced women. I cannot understand the indifference of parents and guardians to the physical and moral well-being of their girls. They do not seem to realise that the institutions where very young probationers are welcomed are just the worst possible places for impressionable girls. The patients view of the matter is but little thought of, or surely they would not be trusted in such untried, incompetent hands. I hope you will let us have some more articles on practical subjects when you can spare space for them.

### COMFORTABLE CONTRIBUTIONS.

"W. R." writes: In answer to the appeal made on behalf of the children visited by the Glasgow Sick Poor Association Nurses came most liberal responses. Parcels of toys were received from *Dundee People's Journal*, Sunbeam Club, from Glasgow Children's Ministering League, and many others. A Christmas tree was adorned at the Home, and about forty little guests (surgical cases) were wheeled or carried to it. The nurses carried gifts next day to seventy other children too ill to be present. An anonymous gift of £5, and smaller sums amounting to £1 19s., enabled various purchases of fowls, rabbits, tea, sugar, and coals to be made, and these were distributed to adult patients. The nurses were repaid for their trouble, and the donors amply rewarded, by the grateful looks and words with which the kindnesses were accepted.

### ENDOWED BED.

"F. L. E." writes: As others may be puzzled besides our good Editor, may I explain what I mean by "nurses taking part in the scheme to have first right to occupancy"—viz., in the event of a nurse-collector nominating a non-collecting nurse for the benefit, should there be any nurse-collector on the application list, she should have the prior claim. In the more improbable event of the whole thousand requiring the bed at the same time, then the sweet spirit of self-denial must decide for her who needs it most.

NURSE M. ELLIOTT writes: I shall be pleased to collect £1 or upwards for bed, if collecting form is sent to address, which I enclose.

"NURSE MARY" writes: It is pleasant to find that the interest in our endowed bed is keeping up so well. I do not see any objection to annual subscriptions, but "F. L. E.'s" scheme is not a bad one, and I shall be glad to hear what other nurses have to say. I hope you will get a lot of letters on this subject.

⁂ ALL nurses wishing to collect are invited to send their name and address, and the amount, to the Editor of THE HOSPITAL, 140, Strand, W.C., on or before 14th March next.

### "CHARITY THINKETH NO EVIL."

"A LICENSED LAY-READER" writes: The anathema passed by the Rev. Mr. Jones is, to say the least, disgraceful in the extreme and sadly uncharitable. If he could not refrain from speaking of "curses," silence surely would have been more becoming. His experience of trained nurses must be very limited. The present writer, from large personal experience, has had the greatest cause to sing the praises of trained nurses very highly, and trusts they may, as a noble band of ministering servants, ever be tenderly cared for and richly blessed.

### Appointments.

WORCESTER.—Miss A. M. Harper has been appointed Lady Superintendent of the City and County Nursing Institution. Miss Harper was trained at Guy's Hospital, and afterwards held the post of Lady Superintendent at Wirrall Children's Hospital.

GILDERSOME, NEAR LEEDS.—Miss Holloway, who held the position of Lady Superintendent at the Nursing Institution at Worcester for over seven years, has just been appointed Matron of the New House of Recovery and Convalescent H me at Girdlesome.

### Medico-Psychological Association.

The following was the examination for certificate of proficiency in nursing, November, 1892:—

1. What are the chief ways by which a patient may attempt suicide, and how would you guard against them?
2. If a large lump of solid food enters the upper air passages of a patient what symptoms would you expect to result, and what would you do?
3. In what class of patients are fractured ribs most liable to occur? What precautions would you take to prevent them?
4. Describe the ordinary severe attack of epilepsy and also the milder attack. What would you do with regard to an epileptic patient during an attack?
5. Mention some of the more common delusions to which melancholic patients are subject.
6. What points would you be careful to attend to in relation to a patient who had had a set of severe repeated convulsions?
7. Give a *brief* account of the disease known as General Paralysis, mentioning the chief symptoms which are met with in the different stages.
8. What are the chief conditions which would lead you to make an immediate and urgent report about a patient?
9. Describe how you would make a poultice, and how you would apply hot fomentations.
10. How would you do with regard to a patient who appears to have fainted?
11. What are the rules which ought to be followed when patients are being bathed?
12. What *general* measures should an attendant take for the prevention of bedsores?

### Minor Appointments.

Miss Kathleen D. Aird has been appointed Sister in Her Majesty's Indian Nursing Service, and sails in H.M.S. *Euphrates* on February 16th. Miss Aird was first trained at the Evelina Children's Hospital, and afterwards for three years at the London Hospital. Her certificates and testimonials are good, and we wish her success in her new work.

### Notes and Queries.

### Queries.

(13) *The Queen's Letter.*—Could you tell me where I could get a copy of the letter. Many nurses would be glad to buy it?—*A Mildmay Nurse.*

(14) *Where to Get Trained.*—Can any one tell me where a person with considerable knowledge of the housekeeping and management of an institution can get training as a nurse? She wishes to fit herself for the matronship of a convalescent home.—*M. B.*

(15) *Examination Questions.*—Will the editor let us have a question on cholera nursing by and by?—*Nurse Annette.*

(16) *Endowed Bed.*—I like your correspondent's idea of the name of the bed being the Hospital Endowed Bed. Please say what places are suggested as more convenient than Ventnor.—*L. M. N.*

(17) *Chicago.*—Is there any chance of nurses getting sent to the exhibition? Many of us want to know about this.—*Not a B.N.A.*

(18) *How to Become a Nurse.*—Will you kindly inform me where I can get the book which I saw reviewed in The Hospital.—*Gertie.*

### Answers.

(13) *The Queen's Letter* (Mildmay Nurse).—They are reproduced by Messrs. Raphael Tuck and Sons, 72, Coleman Street, E.C.

(16) *Endowed Bed* (L. M. N.)—Hastings and Eastbourne have been named, but some persons seem to fancy that a pretty country place would be equally liked. We hope all subscribers will give us their opinions on this point.

(17) *Chicago* (Not a B.N.A.)—If you had read page cxix. of the Nursing Supplement of January 14th, you would have seen that the Congress is organised by the citizens of the United States, and not by English people. We think the paragraph referred to will answer your question fully.

(18) *How to Become a Nurse* (Gertie).—The book is 2s. 6d., and is published by the Scientific Press, 140, Strand.

### Wants and Workers.

A home wanted for a person unable to walk; her general health is good, but she cannot leave her room. She is clever in all sorts of needlework, both plain and fancy, and would work in return for board and lodging. Particulars of *Miss Purchase, 2, Westbrook Cottages, Margate.*

### LEISURE THOUGHTS.

In almost every day of our lives there are moments when, our necessary work being done, we can let our thoughts travel as they please, and indulge ourselves in their wanderings without restraint. If this be the case in ordinary life, how much more time is there for roving thoughts during sickness, when, the body being shut off from its usual pursuits, the mind turns in on itself for amusement. What a variety of ideas would pass before us if we could see into our neighbours' minds. Under such circumstances, some people would be found simply listless, hardly conscious that they were thinking at all; others would be building castles in the air, the ambitious would ponder how they could get on in the world, the vain would dwell on any scraps of praise they had received. Others would be full of their grievances; again others of their anxieties; some would ride away happily on their hobbies, while others, unhappily, think of their health, must we, alas, credit some with indulging in thoughts which lead to shame and ruin. It is by these leisure thoughts that our characters are formed; we should therefore be careful to guide them towards subjects which will influence us for good and not for evil. The power of habit is so strong that, if we indulge in wrong thoughts the expression of our faces will betray us, much as we may fancy we can hide our real feelings, and our tongue will follow suit, for "out of the fulness of the heart the mouth speaketh."

So certain is this, that in all times philosophers and teachers have warned men "to cherish happy, bright, and gentle thoughts" because "our manners will depend very much upon the quality of what we frequently think on." Again, a modern writer has, in a powerful story, shown how a good man may turn into a bad one against his better wishes simply by giving way to evil thoughts which he at first could have controlled, but which in the end made him their slave.

These are true utterances as far as they go, but how should we deal with ourselves so that a happy expression may dwell on our faces? If we turn to our Bibles we shall find what it says on the subject. St. Paul counsels the Philipians as follows: "Whatsoever things are true, whatsoever things are honest, whatsoever things are just, whatsoever things are pure, whatsoever things are lovely, whatsoever things are of good report, if there be any virtue, and if there be any praise, think of these things." He by no means limits us to what are called sacred or religious subjects; art, literature, and science may all be the subjects of consideration, but they must be pure, generous thoughts about them, thoughts that make life pleasant to ourselves and helpful to others. They should be truthful, that is frank and straightforward; they should be just in giving credit for all the good we hear and see in persons and things; especially should they be pure as leading to the eternal purity of Jesus Christ.

### Nursing and Nurses' Institutions in Glasgow.

BY OUR SPECIAL COMMISSIONER.

THE new nursing home at the Western Infirmary, Glasgow, contains the largest and most airy bed-rooms for the accommodation of the head nurses which we have seen anywhere. Each nurse has a separate bed-room, which contains a fire-place and window, important points often overlooked by architects and hospital committees in settling the details of nurses' homes. The home is approached by a handsome conservatory, which is supplied with plants and ferns from the Botanic Gardens adjacent. Here the social functions of the hospital are usually conducted, as the conservatory makes an excellent reception-room, and is a pleasant place to take afternoon tea in. In the home is a corridor pavilion of two storeys, the nurses' rooms being right and left, together with a fair proportion of baths, lavatories, and other conveniences. The bed-rooms do not contain wash-hand basins, because, it was explained, they are unable to provide a staff to do housemaid's work, and each nurse, being responsible for her own room, prefers to use the lavatory and bath-room at somewhat less inconvenience than would result from the extra work attending washstands, &c., in the bed-rooms. Lavatories seem to be a pretty general practice in nurses' homes in Scotland, and may account in a measure for the lessened cost of administration. At the end of the main corridor there is an excellent reading-room, containing technical books for the nurses' use, which is used as a study either for probationers or others who are reading for examination. There is also a truly splendid recreation-room, handsomely furnished, and containing every comfort, and some really good pictures. This recreation-room is lighted on one side by a window, which opens out into a little conservatory running the whole length of the room, so obviating the prospect of a bare wall. At one end it looks out on to a lawn-tennis ground and garden, where the nurses take their recreation during their hours off duty. Altogether this home is entitled to rank as a type of the best buildings of its class, marking a distinct advance in construction in many ways.

This may also be said of the Nursing Home at the Victoria Hospital, where great pains have been taken to make the nurses comfortable and happy. The recreation-room is a fine apartment, which might contain a few more easy chairs, whilst the bed-rooms and their furniture are all that could be desired.

The Nurses' Home at the Royal Infirmary is a fine building. The rooms it contains are on the whole satisfactory, and although the recreation-room will not bear comparison with that at either the Western or Victoria Infirmaries, it is a comfortable apartment, and no doubt fulfils the purpose to which it has been put. The Royal possesses the largest box-room, and one that is better organised than any we have met with elsewhere. Another feature of the nurses' homes connected with the hospitals of Glasgow is the excellent libraries they contain. In this respect they are far in advance of most of the southern hospitals.

The Higginbotham Memorial Home has only been opened a few months. Its structural features and the character of its decoration are striking, and on the whole good. There is a large staff of district nurses attached to this Home, who do most excellent work amongst the sick poor of Glasgow. Fifteen certificated nurses, working under the supervision of the district superintendent, are in daily attendance at the homes of the poor. The whole of the poorer districts in Glasgow are covered by the organisation; the average number of visits paid daily by each nurse is 16. A nurse leaves the Home at half-past nine every morning, and remains on duty till half-past four. Taking her luncheon with her, she returns to dinner at five. In addition to the ordinary daily visits special ones are paid to serious cases in the evening and on Sundays. Besides the district nurses the association has in its employment several trustworthy women who take charge of the more serious cases during the night, acting under the directions of a district nurse, who is herself responsible to the medical attendant. As all the work is done gratuitously thank-offerings are accepted occasionally, and where the patient's means allow a weekly charge is made for night attendance. In specially urgent cases nourishment is supplied by the association, but the funds at its disposal are too limited to enable this to be done to any great extent. Having had the pleasure of an interview with the whole of the district nurses, we are in a position to state, that they are most intelligent, capable, and devoted women. The Higginbotham Home is so constructed as to enable district nurses to return to it by a side door, to enter an isolation room, where they can change their things and so minimise any risk of infection or other disagreeables. The staff of nurses at the Higginbotham Home, 218, Bath Street, Glasgow, are so greatly in demand, that it very frequently happens that the whole staff is out for months at a time, so that it is difficult to supply the demands which may be made upon it.

In a previous article we referred to the new departure in the training of nurses at the Royal Infirmary, Glasgow. Experience has shown that owing to the heavy work in the wards, it is not easy to arrange for attendance at lectures, or to ensure that the nurses shall attend regularly, and at a time when they can be spared from their work. It is, therefore, satisfactory that after due consideration an attempt is about to be made to meet this difficulty by providing an extra mural school which all intending probationers will have to attend for a three months' course before being entered on the hospital books. During this time they will receive instruction in elementary anatomy, physiology, and the practical duties of a nurse, devoting their whole time and attention to these studies as opposed to the practice of their calling. It is hoped and believed that probationers will thus acquire a better knowledge of the elements of their calling than they could reasonably be expected to do under the old system. We shall watch the experiment with interest, and shall be glad to hear more about it after it has been sometime in actual work.

There is evidence of a distinct advance in nursing matters at Glasgow all along the line. It is now generally admitted that the outcry which was raised at the Royal Infirmary has resulted in marked improvement in many directions, and so has done great and substantial good. We believe that the nursing arrangements and the accommodation and training which the probationers receive there are thoroughly efficient, and that they fulfil every reasonable requirement. It is certainly satisfactory that public opinion has so ripened on this question, that throughout the country evidence is forthcoming that everywhere the managers of institutions where nurses are trained have become thoroughly impressed with the importance of this branch of the work they undertake and have so provided that it shall be conducted with due regard to efficiency, the comfort of the probationers, and the nursing staff.

### Where to Go.

CONCERT at Westminster Town Hall in aid of funds of Grosvenor Hospital for Women and Children, on February 6th. Patroness H.R.H. Duchess of Fife.

LECTURES by Dr. Bridges every Sunday in January and February, at 7 p.m., Newton Hall, Fetter Lane, on "Health, Physical, Intellectual, and Moral."

## Figures and Tables

**Figure f1:**
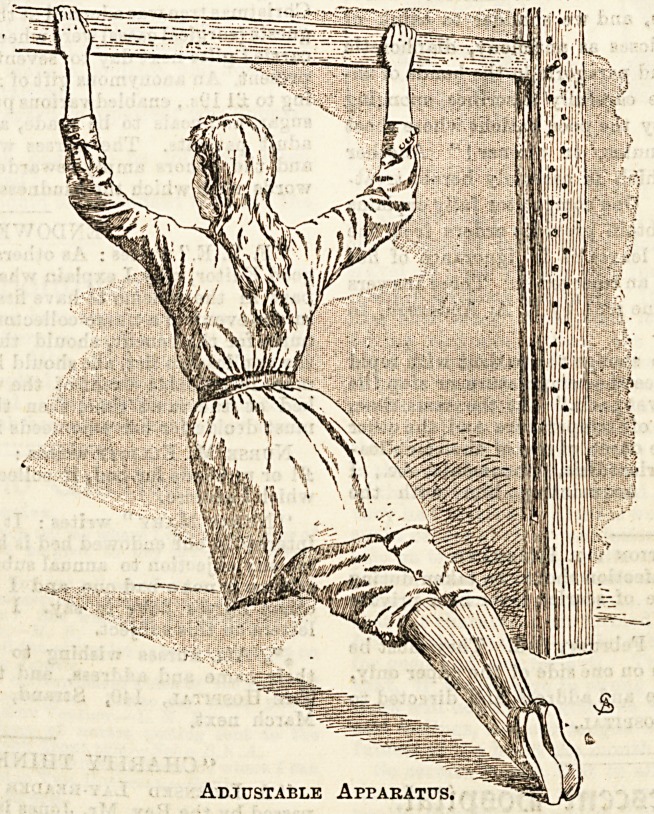


**Figure f2:**